# Context matters: faculty norms on binge drinking relate to binge drinking behaviour in higher education

**DOI:** 10.1186/2049-3258-73-S1-P13

**Published:** 2015-09-17

**Authors:** Joris Van Damme, Anne Hublet, Bart De Clercq, John McAlaney, Guido Van Hal, Johan Rosiers, Lea Maes, Els Clays

**Affiliations:** 1 Ghent University, Gent, Belgium; 2 Bournemouth University, Bournemouth, United Kingdom; 3 Antwerp University, Antwerp, Belgium; 4 Association for Alcohol and other Drug problems, Brussels, Belgium

## Background

In higher education binge drinking is an important problem. To target binge drinking in students, studying the social context of students is necessary. Faculties are social contexts in which students behave, but little is known about how faculty binge drinking norms relate to monthly binge drinking. In this study, this relationship is investigated in addition to known personal determinants.

## Methods

Data were collected from 7,181 students in 22 faculty-level units, using an anonymous online survey. Multilevel analyses were used to investigate the relationship of both individual-level determinants (i.e., perceived binge drinking norms and social drinking motives) and faculty-level binge drinking norms, with monthly binge drinking.

## Results

Almost two-third (62.2%) of the sample was female and the mean age was 21.06 (SD = 2.85) years. In males, significant faculty-level variance in monthly binge drinking was found. At faculty-level only faculty binge drinking norms about male students showed a positive relationship (OR = 2.586; 95%CI = [1.025, 6.522]). At individual level both perceived binge drinking norms about male and female students, and social drinking motives positively related to monthly binge drinking. In females no significant faculty-level variance was found. Only individual-level determinants (i.e., perceived binge drinking norms and social drinking motives) positively related to monthly binge drinking. No cross-level interactions were found.

## Conclusion

Faculties are especially in men relevant environmental structures and networks to take into account besides individual determinants when targeting binge drinking in higher education.

